# Systemic Mastocytosis: Molecular Pathophysiology, WHO Diagnostic Framework, and *KIT*-Directed Targeted Therapies

**DOI:** 10.3390/cancers18142205

**Published:** 2026-07-08

**Authors:** Caterina Alati, Maria Bruna Greve, Gaetana Porto, Giorgia Policastro, Erica Bilardi, Giovanna Utano, Laura Giordano, Martina Pitea, Massimo Martino

**Affiliations:** Hematology and Stem Cell Transplantation and Cellular Therapies Unit (CTMO), Department of Hemato-Oncology and Radiotherapy, Grande Ospedale Metropolitano “Bianchi-Melacrino-Morelli”, 89133 Reggio Calabria, Italyerica.bilardi@ospedalerc.it (E.B.);

**Keywords:** systemic mastocytosis, *KIT* D816V, mast cell disease, targeted therapy, *KIT* inhibitors

## Abstract

Background: Systemic mastocytosis (SM) is a clonal mast cell (MC) neoplasm driven by somatically acquired activating mutations in the *KIT* receptor tyrosine kinase (CD117), resulting in pathological accumulation of morphologically atypical MCs in extracutaneous organs. The *KIT* D816V substitution is detectable in over 95% of cases by high-sensitivity next-generation sequencing (NGS) or allele-specific PCR. This gain-of-function variant confers ligand-independent receptor autophosphorylation, leading to constitutive activation of downstream signaling cascades that promote MC progenitor survival, clonal expansion, and resistance to apoptosis. Co-occurring somatic mutations in *TET2*, *SRSF2*, *ASXL1*, *CBL*, and *RUNX1*, increasingly identified in the context of clonal hematopoiesis of indeterminate potential, are associated with more aggressive phenotypes and independently confer adverse prognostic impact. Results: The 2022 WHO classification delineates indolent forms from advanced-phase SM, aggressive SM, SM with an associated hematologic neoplasm (SM-AHN), and MC leukemia, which produce progressive end-organ damage through both neoplastic tissue infiltration and uncontrolled mediator release. Formal diagnosis requires integration of histological criteria (multifocal bone marrow MC aggregates of ≥15 cells), immunophenotypic aberrancies (CD25, CD2, and/or CD30 coexpression on MCs by flow cytometry or immunohistochemistry), biochemical markers (baseline serum tryptase ≥ 20 ng/mL), and molecular confirmation of *KIT* D816V or equivalent pathogenic *KIT* mutation. The development of type I *KIT* inhibitors with selectivity for the D816V-mutant conformation has fundamentally restructured the therapeutic field of advanced SM. Conclusions: This review provides a thorough synthesis of SM pathobiology, WHO-defined diagnostic and classification criteria, validated prognostic tools, and the developing landscape of *KIT*-directed and combination therapies, with direct translational relevance for specialist practitioners managing this heterogeneous myeloid neoplasm.

## Simple Summary

Systemic mastocytosis (SM) is a clonal mast cell (MC) neoplasm driven by somatically acquired activating mutations in the *KIT* receptor tyrosine kinase (CD117), resulting in pathological accumulation of morphologically atypical MCs in extracutaneous organs. The *KIT* D816V substitution is detectable in over 95% of cases by high-sensitivity next-generation sequencing (NGS) or allele-specific PCR. The development of type I *KIT* inhibitors with selectivity for the D816V-mutant conformation has fundamentally restructured the therapeutic field of advanced SM. This review provides a thorough synthesis of SM pathobiology, WHO-defined diagnostic and classification criteria, validated prognostic tools, and the developing landscape of *KIT*-directed and combination therapies, with direct translational relevance for specialist practitioners managing this heterogeneous myeloid neoplasm.

## 1. Introduction

Mastocytosis is a neoplastic disorder characterized by clonal mast cell (MC) accumulation in one or more organs. It is classified as cutaneous mastocytosis (CM), which is limited to the skin; systemic mastocytosis (SM), which involves extracutaneous organs with or without skin involvement; or the rare mast cell sarcoma (MSC) [1]. While both SM and mast cell activation syndrome (MCAS) present with overlapping symptoms due to mast cell mediator release, SM is a distinct clinical entity [2]. Idiopathic MCAS derives from hyperactivation of non-clonal mast cells and lacks the defining histopathological and molecular features of SM [3]. SM encompasses a heterogeneous clinical and biological spectrum rather than a single disease. Indolent SM (ISM) is the most prevalent form in adults and is associated with a near-normal prognosis. In contrast, aggressive SM (ASM), SM with associated hematological neoplasm (SM-AHN), and mast cell leukemia (MCL) are associated with increased morbidity and mortality [4]. A comprehensive understanding of the molecular basis, diagnostic modalities, subclassification, and therapeutic strategies is vital for specialist clinicians. Clonality in SM is established at the molecular and immunophenotypic level, typically by detecting a somatic activating *KIT* mutation (most often D816V) in lesional mast cells and/or by demonstrating an aberrant, disease-restricted mast cell immunophenotype (CD25 and/or CD2 co-expression) by flow cytometry or immunohistochemistry; X-chromosome inactivation (HUMARA) assays were used historically to demonstrate mast cell monoclonality before molecular methods became standard.

## 2. Pathophysiology

**Figure 1:**
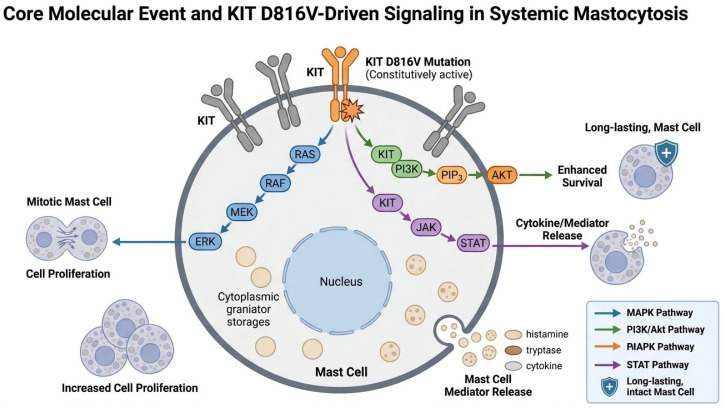
Core Molecular Event and *KIT* D816V-Driven Signaling in Systemic Mastocytosis.

The central molecular event in SM is a somatic gain-of-function mutation in the *KIT* gene. *KIT* encodes the *KIT* receptor tyrosine kinase, also known as CD117 or stem cell factor receptor (Figure 1) [5]. *KIT* is the focus of SM pathobiology both because activating *KIT* mutations are the most frequent molecular abnormality in SM, present in over 90% of patients, and because *KIT* D816V was the first molecular lesion identified in SM and has since been shown to be necessary and sufficient to drive ligand-independent mast cell proliferation and survival, making it the central, disease-defining oncogenic driver rather than merely the most common incidental finding.

Under physiological conditions, stem cell factor (SCF) binds to *KIT*, activating the receptor and downstream signaling through the JAK/STAT, MAPK/ERK, PI3K/AKT, and PLCγ pathways. These pathways regulate mast cell proliferation, survival, and migration, each with a partially distinct contribution: JAK/STAT signaling drives proliferation and pro-survival gene transcription; MAPK/ERK signaling promotes proliferation and cytokine production; PI3K/AKT signaling supports survival by inhibiting apoptosis; and PLCγ signaling regulates degranulation and calcium-dependent mediator release. Over 90% of patients with mastocytosis harbor a *KIT* gene mutation, with the D816V substitution in exon 17 present in 85–90% of adults [6]. This mutation leads to ligand-independent receptor activation. The D816V substitution lies within the *KIT* activation loop (exon 17) and destabilizes the autoinhibited conformation of the kinase domain, locking the receptor in an active, ligand-independent state; this conformational shift alters the ATP-binding pocket relative to wild-type *KIT*. Consequently, first-generation tyrosine kinase inhibitors (TKIs) such as imatinib, which require the inactive conformation for high-affinity binding, are ineffective because they cannot bind the altered protein conformation, whereas type I inhibitors such as avapritinib and bezuclastinib were rationally designed to accommodate the active-state conformation. Constitutive *KIT* signaling promotes expansion of clonal mast cells, which accumulate in the bone marrow, liver, spleen, gastrointestinal tract, and other extracutaneous organs. This accumulation results in the release of vasoactive, proinflammatory, and fibrogenic mediators, including histamine, prostaglandin D2, heparin, tryptase, leukotrienes, and cytokines such as IL-6 and TNF, which together produce the systemic manifestations of SM [7,8].

Under normal physiological conditions, mast cells are tissue-resident innate immune cells that arise from bone marrow progenitors and mature locally in the skin, gastrointestinal tract, respiratory mucosa, and other organs. They contribute to host defense against parasites and certain bacteria, participate in IgE-mediated allergic responses, and play recognized roles in tissue remodeling, angiogenesis, and wound healing through the coordinated release of preformed and newly synthesized mediators. In SM, this normal physiological repertoire of mediator release is preserved but becomes pathologically amplified and dysregulated as a consequence of clonal mast cell expansion, explaining why SM manifests principally as an exaggeration of otherwise physiological mast-cell-driven processes.

Chronic and episodic release of mast cell mediators results in a broad spectrum of symptoms, some of which are debilitating [9]. Cutaneous manifestations, primarily attributable to histamine, include pruritus, urticaria, flushing, and angioedema. Gastrointestinal symptoms, such as abdominal cramping, nausea, vomiting, and diarrhea, arise from the actions of histamine and prostaglandins on the gut mucosa. Cardiovascular instability, presenting as presyncope, tachycardia, and anaphylaxis, is mediated by vasodilatory and anaphylactogenic substances. Neurocognitive symptoms, including cognitive impairment, poor concentration, and headaches, are also recognized, although their basic mechanisms remain incompletely understood.

In advanced stages, mast cell infiltration of organs leads to structural damage that is independent of mediator-related symptoms [10]. Complications may include cytopenias due to bone marrow infiltration, hepatosplenomegaly, portal hypertension, and osteoporosis. Bone involvement results from both direct mast cell invasion and the inhibitory effects of heparin on osteoblast function. While all SM subtypes negatively affect quality of life, advanced disease has the greatest impact.

## 3. Clinical Presentation and Differential Diagnosis

Before comparing age-related presentations, it is important to distinguish clearly between cutaneous mastocytosis (CM) and systemic mastocytosis (SM). CM is confined to the skin, with no histological or molecular evidence of extracutaneous mast cell infiltration, and it predominates in children. SM, by definition, requires demonstration of clonal mast cell involvement in one or more extracutaneous organs (most often the bone marrow) fulfilling the WHO/ICC diagnostic criteria detailed in Section 4, and it predominates in adults. Because Table 1 compares the typical mastocytosis phenotype across age groups, it necessarily contrasts predominantly cutaneous pediatric-onset disease with predominantly systemic adult-onset disease, rather than CM versus SM within a single age group.

SM in adults and children is a different entity (Table 1) [11].

In adults, indolent SM (ISM) is the most common form. It is chronic and does not resolve. In adults, small monomorphic maculopapules, known as urticaria pigmentosa, are present in over 80% of cases on the thighs and trunk [11]. The Darier sign—whealing and erythema after stroking the lesion—is usually present. Serum tryptase is usually above 20 µg/L in most adults, and *KIT* D816V is found in 85–90% of cases [6]. Pediatric mastocytosis is very different. Cutaneous mastocytosis is most common and usually does not involve systemic organs. The disease frequently resolves spontaneously before adulthood, an observation attributed to the predominantly non-D816V, often non-*KIT*-restricted mutational profile of pediatric skin mast cells, a lower overall mast cell burden confined to the skin, and a presumed self-limited proliferative process during childhood rather than a persistent clonal bone marrow-derived neoplasm as seen in adult SM. Skin lesions are polymorphic and may affect the head and extremities. Tryptase is usually under 20 µg/L. *KIT* mutations are less common and more varied. Anaphylaxis occurs in under 10% of children, which is much lower than in adults (about 50%) [12].

SM presents with episodic and multisystem symptoms. This results in a wide differential diagnosis, and clinicians must consider other mast cell disorders, such as monoclonal and idiopathic MCAS, as well as systemic anaphylaxis from other causes [13]. Other conditions can have similar symptoms (Table 2).

A complete assessment is required to differentiate SM from other conditions presenting with similar symptoms. Diagnostic assessment should include measurement of serum tryptase, skin biopsy when indicated, and bone marrow examination.

## 4. Diagnostic Approach

SM should be suspected in adults presenting with recurrent mast cell mediator release symptoms or anaphylaxis of unexplained etiology, particularly in the setting of a persistently elevated baseline serum tryptase [14]. Adult-onset urticaria pigmentosa confirmed on skin biopsy constitutes an independent indication for further investigation. A baseline serum tryptase ≥ 20 ng/mL, in the absence of chronic renal impairment or concomitant myeloid neoplasm, both of which can independently elevate tryptase, warrants bone marrow evaluation to exclude SM. It is important to note, however, that tryptase thresholds carry imperfect sensitivity: approximately 15% of SM patients present with levels below 20 ng/mL, and up to 10% have values within the normal reference range, underscoring the need for integrating clinical, histological, and molecular criteria rather than relying on biomarker thresholds alone. Hereditary alpha-tryptasemia (HαT), caused by germline duplications or multiplications of the *TPSAB1* gene encoding α-tryptase, is the most frequent cause of a persistently elevated baseline serum tryptase in the general population and must be considered whenever tryptase is elevated, independent of SM [14]. Because HaT can raise baseline tryptase irrespective of mast cell burden and may co-occur with SM, assessment of the underlying *TPSAB1* genotype improves the sensitivity and specificity of tryptase-based diagnostic thresholds and is recommended when an elevated tryptase does not otherwise fit the clinical picture.

The WHO 5th Edition [15] and the International Consensus Classification (ICC) [16] define SM diagnosis using major and minor criteria (Table 3). WHO diagnosis requires one major plus at least one minor criterion. ICC requires one major or three or more minor criteria. The ≥15 mast cell aggregate threshold for the major criterion and the associated minor criteria thresholds were empirically derived and iteratively refined through consensus by the WHO and ECNM working groups on the basis of comparative histopathological and outcome data distinguishing SM from reactive mast cell hyperplasia and from CM [15,17]; they are not derived from a single index study but represent an evolving consensus standard periodically re-validated as diagnostic experience accumulates.

When SM is suspected, several steps are needed. These include bone marrow biopsy with MC immunophenotyping and molecular testing for *KIT* D816V. Additional *KIT* sequencing is done if required. In *KIT* D816V-negative patients with eosinophilia, screen for the *FIP1L1*::*PDGFRA* fusion.

Real-world diagnosis is often complicated by atypical presentations that do not fit the classic profile. A meaningful minority of patients with confirmed SM have a normal baseline serum tryptase, particularly in BMM and in some ISM cases with low mast cell burden, so a normal tryptase does not exclude SM in a patient with a compatible clinical picture. Similarly, mast cells can show an atypical immunophenotype (e.g., weak or absent CD25 expression) in a subset of patients, and bone marrow mast cell aggregates can be sparse or spindle-shaped rather than the classic round, atypical morphology, increasing the risk of a false-negative biopsy if an experienced hematopathologist is not involved. In such atypical cases, repeat or multisite bone marrow sampling, sensitive allele-specific *KIT* D816V PCR performed on peripheral blood or bone marrow, and correlation with clinical mediator-release symptoms and imaging are recommended before SM can be confidently excluded.

## 5. Disease Classification and Subtyping

Accurate subclassification is essential (Table 4) [17]. It determines prognosis and guides treatment. SM is either non-advanced or advanced based on B- and C-findings. B-findings indicate worsening SM, but no organ damage yet. They include a high mast cell burden, signs of myeloproliferation and/or myelodysplasia in a non-mast-cell lineage not fulfilling criteria for an associated hematologic neoplasm (AHN), enlarged liver, enlarged spleen, or swollen lymph nodes. C-findings mean SM has caused organ damage. These signs include low blood counts due to mast cell infiltration of the bone marrow, an enlarged and poorly functioning liver, difficulty absorbing nutrients, bone breakdown with fractures, or a very large spleen that lowers blood cell counts.

Differentiating between non-advanced and advanced SM is critical for guiding treatment decisions. Patients with ISM or SSM are initially managed with antimediator therapy, and, more recently, selective *KIT* inhibition has been introduced for symptom control. In contrast, patients with advanced SM require cytoreductive therapy to reduce mast cell burden.

## 6. Therapies for Systemic Mastocytosis

For many patients with indolent SM, symptom-directed antimediator therapy (Section 13) provides adequate long-term disease control, and pharmacologic cytoreduction is not required. The discussion that follows focuses on *KIT*-directed targeted therapies, which are the mainstay for advanced SM and are increasingly used in indolent or smoldering SM when symptoms remain uncontrolled despite optimized antimediator treatment.

**Figure 2:**
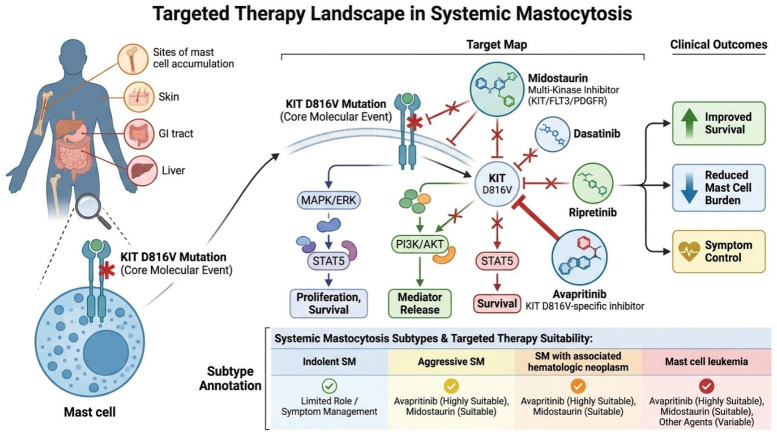
Targeted Therapy in Systemic Mastocytosis.

The therapeutic field of systemic mastocytosis (SM) has been fundamentally transformed by the molecular characterization of the *KIT* D816V somatic mutation, which is present in over 90% of cases and constitutively activates *KIT* signaling, driving neoplastic mast cell proliferation and survival [18] (Figure 1). This discovery established the rationale for selective kinase inhibition as the central therapeutic strategy in advanced SM (Figure 2) (Table 5).

## 7. Midostaurin

Midostaurin (PKC412) was the first targeted agent globally approved for advanced SM, including aggressive SM (ASM), SM with associated hematologic neoplasm (SM-AHN), and mast cell leukemia (MCL) [36,37]. As a multikinase inhibitor active against *KIT* D816V, *FLT3*, PDGFR, and VEGFR, midostaurin demonstrated meaningful reductions in mast cell burden, serum tryptase levels, splenomegaly, and symptom severity in the pivotal CPKC412D2201 trial, achieving an overall response rate of approximately 60% in advanced SM [20]. Its approval represented a paradigm shift from purely cytoreductive or symptomatic approaches toward mutation-targeted therapy [38]. In the pivotal open-label CPKC412D2201 trial, 116 patients with advanced SM (aggressive SM, SM-AHN, and MCL) received midostaurin 100 mg twice daily; the overall response rate by prespecified criteria was approximately 60%, with responses observed across all three advanced subtypes, and median overall survival exceeded two years in responders [20]. The most common adverse events were low-grade gastrointestinal toxicities—nausea, vomiting, and diarrhea—occurring in the majority of patients and generally manageable with antiemetics and dose modification; myelosuppression and hepatotoxicity were also reported and required monitoring [20,38]. Ten-year median follow-up confirmed durable responses in a subset of patients without new safety signals [37]. Unlike avapritinib, midostaurin is a multikinase inhibitor with only modest selectivity for *KIT* D816V, which underlies its broader off-target toxicity profile but does not require the REMS-type monitoring associated with more potent, selective type I inhibitors.

## 8. Imatinib

Imatinib retains a narrow but defined role in the rare subset of SM patients lacking the *KIT* D816V mutation, particularly those harboring wild-type *KIT* or transmembrane/juxtamembrane domain mutations, where it can achieve meaningful responses [19]. It has no meaningful activity against *KIT* D816V and is not appropriate for most SM patients.

## 9. Avapritinib

Avapritinib is a selective, potent inhibitor of *KIT* D816V (and *PDGFRA* D842V) that has changed the treatment framework for SM [39]. Unlike imatinib, avapritinib was specifically designed to bind the activated conformation of the mutant *KIT* kinase domain, overcoming the structural barrier to inhibition posed by the D816V substitution. In advanced SM, avapritinib showed strong responses in the phase II PATHFINDER trial [21,22]. Across all advanced SM subtypes, it produced sustained reductions in mast cell burden, serum tryptase, and *KIT* D816V variant allele frequency (VAF), with overall response rates well above prior standards of care.

The pivotal phase II/III PIONEER trial (N = 212) evaluated avapritinib 25 mg daily versus placebo in patients with ISM and moderate-to-severe symptom burden (Total Symptom Score ≥ 28) despite two or more antimediator drugs [23]. Key findings at 24 weeks included Total Symptom Score reduction of ≥50%: 25% of avapritinib-treated patients vs. 10% with placebo (*p* = 0.005); serum tryptase reduction ≥ 50%: 54% vs. 0% (*p* < 0.001); *KIT* D816V VAF reduction ≥50%: 68% vs. 6% (*p* < 0.001); and bone marrow mast cell burden reduction ≥50%: 53% vs. 23% (*p* < 0.001). Avapritinib also produced meaningful reductions in skin lesion area across multiple body regions at 24 weeks, with mean reductions ranging from approximately 31% to 57%, compared with minimal change with placebo. Long-term data from the PIONEER open-label extension (data cutoff: September 2024) demonstrate durable and deepening benefit: the mean Total Symptom Score change from baseline was −17.51 at Week 96 and −20.07 at Week 144 [24]. Improvements were seen across gastrointestinal, skin, and neurocognitive symptom domains, with parallel improvements in mastocytosis-specific quality-of-life scores.

Avapritinib is generally well tolerated in ISM at the approved 25 mg daily dose [23,24]. The most common adverse reactions (≥10% incidence in ISM) are periorbital/eye edema, dizziness, peripheral edema, and flushing. In advanced SM at higher doses, edema, diarrhea, nausea, and fatigue/asthenia are more prominent (≥20% incidence). Key warnings and precautions include intracranial hemorrhage, observed in 2.9% of patients with GIST or advanced SM in clinical trials; no events occurred in ISM patients in PIONEER. Fatal events were reported in <1% of trial participants overall. Cognitive effects are dose-dependent and generally reversible on withholding or reducing the drug. Patients should be advised to avoid direct UV exposure. Embryofetal toxicity: effective contraception is mandatory. Per guidelines, cladribine and tyrosine kinase inhibitors are not recommended during pregnancy.

## 10. Investigational KIT Inhibitors

Two additional selective *KIT* inhibitors are in advanced clinical development and show notable promise.

The SUMMIT clinical trial is a multi-part, randomized, double-blind, placebo-controlled Phase 2 study evaluating bezuclastinib in patients with indolent or smoldering systemic mastocytosis whose symptoms remain uncontrolled by best supportive care [31,32]. Treatment with a 100 mg dose demonstrated rapid, clinically meaningful, and durable reductions in overall patient symptom severity [31,32]. These improvements were measured by the validated Mastocytosis Symptom Severity Daily Diary (MS2D2) scale. Patients experienced significant and deep reductions in standard diagnostic markers of mast cell burden [32]. This includes dramatic drops in serum tryptase levels, bone marrow mast cell burden, and *KIT* D816V variant allele frequency (VAF). The APEX clinical trial is an open-label, registration-directed Phase 2 study targeting life-threatening subtypes of SM, including aggressive SM (ASM), SM with an associated hematologic neoplasm (SM-AHN), and mast cell leukemia (MCL) [33,34]. Long-term data from the trial demonstrated a 24-month progression-free survival (PFS) rate of 82% alongside objective reductions in total disease burden [33,34]. Most treatment-emergent adverse events were low-grade, highly manageable, and reversible. Common mild side effects included hair color changes, alterations in taste (dysgeusia), nausea, and transient elevations in liver enzymes. Unlike older-generation TKIs, bezuclastinib distinguishes itself through precise structural engineering [35]. It avoids off-target inhibition of closely related kinases such as PDGFRα, PDGFRβ, and CSF1R. Sparing these pathways markedly decreases the likelihood of toxicities like severe edema, pleural effusions, and bleeding events. Bezuclastinib was designed specifically not to cross the blood–brain barrier [35]. This low central nervous system penetration successfully eliminates the severe cognitive impairments and intracranial hemorrhage risks associated with alternative agents like avapritinib.

Elenestinib (BLU-263) is a novel, investigational, oral, next-generation tyrosine kinase inhibitor that potently and selectively inhibits *KIT* D816V with limited central nervous system penetration and pharmacokinetics that support once-daily dosing [25,26,27]. HARBOR is a randomized, double-blind, placebo-controlled, Phase 2/3 study comparing the efficacy and safety of elenestinib + symptom-directed therapy (SDT) with placebo + SDT in participants with ISM whose symptoms are not adequately controlled by SDT [29,30]. Part 1 was a double-blind, placebo-controlled, dose-finding study that enrolled adult patients with centrally confirmed ISM per WHO criteria and moderate-to-severe symptoms, defined as an ISM Symptom Assessment Form (ISM-SAF) total symptom score (TSS) of at least 28. Patients were randomly assigned to receive placebo plus best supportive care (BSC), elenestinib at 25 mg/day plus BSC, elenestinib at 50 mg/day plus BSC, or elenestinib at 100 mg/day plus BSC. The primary endpoints of Part 1 were safety and pharmacokinetics/pharmacodynamics. Elenestinib at all tested doses demonstrated beneficial effects on disease-related symptoms and biomarkers of mast cell burden in a large, maturing cohort of patients with ISM and moderate-to-severe symptom burden. Key eligibility criteria for Part 2 include an ISM diagnosis and ISM-SAF TSS ≥ 28 at screening. Endpoints include mean change in ISM-SAF TSS from baseline to Week 48 (primary endpoint), achievement of controlled disease (defined as mild TSS), changes in serum tryptase, *KIT* D816V variant allele fraction, bone mineral density, anaphylaxis rate, and safety. Part 2 of HARBOR is ongoing and will deliver the definitive randomized efficacy data, with a particular focus on symptom burden, mast cell biomarkers, bone density, and anaphylaxis reduction [29,30].

Comparative structural selectivity. Avapritinib and bezuclastinib both potently inhibit *KIT* D816V but differ meaningfully in their kinase selectivity and pharmacologic consequences. Avapritinib is a type I inhibitor that also retains substantial activity against *PDGFRA* D842V and wild-type *KIT*/PDGFR family members, and it is brain-penetrant; this broader activity and CNS exposure likely contribute to its efficacy across advanced SM subtypes but are also mechanistically linked to its principal toxicities, including intracranial hemorrhage risk and cognitive effects. Bezuclastinib was engineered for greater structural selectivity for mutant *KIT* D816V over closely related kinases such as PDGFRα, PDGFRβ, and CSF1R and was designed to have low CNS penetration; this narrower off-target profile is associated with a lower observed incidence of edema, effusions, bleeding, and cognitive adverse events in early trials, though cross-trial comparisons are limited by differing patient populations and endpoints, and head-to-head data are not yet available.

## 11. Emerging and Combined Strategies

The recognition that SM frequently co-occurs with clonal hematologic neoplasms—and that additional somatic mutations (*TET2*, *SRSF2*, *ASXL1*, *RUNX1*) confer prognostic significance and may mediate resistance—has prompted interest in combination regimens [30,40]. Trials exploring avapritinib or bezuclastinib in combination with venetoclax, azacitidine, or ruxolitinib are ongoing, aiming at deeper and more durable eradication of the neoplastic clone [41,42]. Allogeneic stem cell transplantation remains the only potentially curative modality in eligible patients with aggressive SM or SM-AHN, though its role is being reassessed in the era of highly effective *KIT* inhibitors [43].

Co-occurring somatic mutations. Beyond *KIT* D816V, mutations in *TET2*, *SRSF2*, *ASXL1*, *CBL*, and *RUNX1*—genes classically associated with clonal hematopoiesis and myeloid neoplasms—are detected in a substantial proportion of patients with SM, particularly SM-AHN and other advanced subtypes, and are markedly less frequent in ISM. *TET2* and *SRSF2* mutations are the most common of this group, and while individually associated with a modest prognostic effect, their co-occurrence with *ASXL1* and/or *RUNX1* mutations (the so-called S/A/R/T genotype when combined with *SRSF2*) identifies a subset of patients with shorter overall and progression-free survival independent of the SM subtype itself. Mechanistically, these mutations are thought to arise in a hematopoietic progenitor upstream of or independent from the *KIT* D816V-mutant mast cell clone, contributing to myelodysplastic and myeloproliferative features of SM-AHN and potentially to resistance to *KIT*-selective inhibition, which targets the mast cell compartment but does not eradicate a co-occurring myeloid clone. Screening for these mutations by targeted next-generation sequencing myeloid panels at diagnosis is therefore recommended in suspected advanced SM to refine prognosis and to identify patients who may benefit from combination strategies or earlier consideration of allogeneic transplantation.

## 12. Cytoreductive Therapy: The Pre-Targeted Era

Prior to the advent of selective *KIT* inhibitors, cytoreductive therapy for advanced SM relied on agents offering limited and often short-lived clinical benefit at the cost of significant toxicity [44]. Interferon-α (with or without corticosteroids) and cladribine (2-CdA) were the mainstays, achieving partial responses in some patients—primarily reductions in splenomegaly, mast cell burden, and symptom severity—but rarely inducing deep molecular remission. Hydroxyurea provided modest cytoreduction in slower-proliferating disease. These agents remain relevant in specific scenarios: rapid debulking requirements, resource-constrained settings, or intolerance to approved targeted therapies, but they have been largely supplanted by molecularly targeted approaches in eligible patients.

## 13. Stepwise Antimediator Therapy

Regardless of SM subtype, symptomatic management of mast cell mediator release forms the essential foundation of treatment and should be initiated in all patients before escalating to cytoreductive or targeted approaches (Table 6) [45,46,47,48].

First-line antimediator therapy centers on combined H1 antihistamines (cetirizine, loratadine, hydroxyzine) and H2 antihistamines (famotidine, ranitidine) to control cutaneous manifestations, urticaria, flushing, pruritus, and gastrointestinal symptoms, including cramping, nausea, and diarrhea. Second-line agents address refractory or multisystem symptoms: cromolyn sodium stabilizes mast cell degranulation along the gut mucosa and reduces systemic mediator burden; proton pump inhibitors manage acid hypersecretion and upper GI complications; and leukotriene receptor antagonists (montelukast) provide additional control of bronchospasm and flushing refractory to antihistamine monotherapy. Prevention and management of anaphylaxis are critical priorities for all SM patients. All patients must always carry two epinephrine auto-injectors, given the unforeseeable and potentially fatal nature of anaphylactic events. Venom immunotherapy is indicated where Hymenoptera sensitization is identified. In refractory or recurrent anaphylaxis, omalizumab (anti-IgE, off-label) has demonstrated success in reducing anaphylactic episodes and mediator-related symptom burden, particularly in patients with elevated baseline IgE or concomitant allergic sensitization. Corticosteroids, systemic or topical, may provide benefit in refractory cutaneous disease, malabsorption syndromes, and ascites refractory to other measures, though chronic use is limited by well-established adverse effects. Bisphosphonates (zoledronic acid preferred) and vitamin D/calcium supplementation address osteoporosis driven by heparin and tryptase release from infiltrating mast cells.

## 14. Expert Opinion

Systemic mastocytosis is a complex mast cell neoplasm that requires a strong index of suspicion for timely diagnosis and appropriate referral to dedicated centers. The heterogeneity of clinical presentations, ranging from indolent to aggressive forms, confirms the importance of a multidisciplinary approach to management. Treatment remains adapted to disease burden and symptom profile. Antimediator therapies address symptomatic relief, while cytoreductive strategies target the underlying neoplastic process. The identification of *KIT* mutations, particularly *KIT* D816V, as central drivers of SM pathophysiology has paved the path for a new generation of precision therapies. Selective *KIT* inhibitors represent a significant advance in the field of treatment, offering the opportunity to modify the disease course rather than merely manage symptoms. Given the chronicity of the disease and the complexity of its management, patients with SM often require concurrent and evolving treatment regimens. Clear, ongoing communication between patients and care providers is therefore not a secondary consideration but a keystone of quality care. Making sure that patients understand their condition, therapeutic options, and the rationale for therapy modifications directly contributes to adherence and outcomes. As the therapeutic armamentarium continues to expand, early referral to expert centers and incorporation of emerging targeted agents will be critical to improving prognosis and quality of life for people living with systemic mastocytosis.

The diagnostic workup should adhere to WHO criteria, incorporating bone marrow biopsy with immunohistochemistry (CD117, CD25, CD2 expression), *KIT* D816V mutational analysis in peripheral blood and bone marrow, and serum tryptase measurement, recognizing that a baseline tryptase ≥ 20 ng/mL is itself one of the four minor diagnostic criteria and a surrogate marker of mast cell burden. Once diagnosis is confirmed, categorization into ISM, smoldering SM (SSM), SM with an associated hematologic neoplasm (SM-AHN), aggressive SM (ASM), or mast cell leukemia (MCL) is critical to determine prognosis and treatment intensity and should be performed in centers with documented expertise in mast cell diseases.

From a clinical management standpoint, referral to a specialized center is not simply recommended but strongly advocated by current international guidelines, including those from the European Competence Network on Mastocytosis (ECNM) and the American Initiative in Mast Cell Diseases (AIM) [11,15]. This is particularly relevant given data from patient surveys, which indicate that diagnostic responsibility is distributed across multiple specialties: hematology/oncology in approximately 32–34% of cases and allergy/immunology in 18–20% [9], highlighting the fragmented and often delayed diagnostic journey patients experience. At the specialized center level, a structured multidisciplinary team should include hematologists, allergists/immunologists, dermatologists, gastroenterologists, endocrinologists, and psychiatrists, each involved in the management of the distinct but interconnected comorbidities that define the clinical complexity of SM.

Mediator-related symptoms, including flushing, urticaria, pruritus, abdominal cramping, diarrhea, and episodic hypotension, are present in most SM patients and represent a main cause of impaired quality of life. Bone disease represents another frequently underdiagnosed comorbidity that requires systematic evaluation and active management [49]. SM-associated osteoporosis is driven by the direct osteolytic effects of mast cell mediators, including histamine, heparin, and various cytokines, and is further potentiated by modifiable risk factors such as vitamin D deficiency, obesity, sedentary lifestyle, and prolonged corticosteroid use. A baseline DXA scan is mandatory in all SM patients at diagnosis, with follow-up assessments guided by clinical risk stratification. Vitamin D supplementation should be initiated in all patients with a deficiency. For patients with high-risk osteopenia (T score approaching −2) or overt osteoporosis (T score ≤ −2), bisphosphonate therapy constitutes the standard of care; in cases refractory to bisphosphonates, a RANK inhibitor (denosumab) represents a valid alternative. Low-dose interferon-alpha (IFN-α) may be considered in selected patients with severe SM-related bone disease unresponsive to conventional therapies. Weight management, dietary optimization, and individualized physical activity programs should be integrated into the management of all SM patients with obesity or sedentary behavior, covering both osteoporosis risk and general metabolic health.

Psychiatric comorbidities, particularly depression and anxiety, are highly prevalent in SM and are substantially underrecognized in routine clinical practice. The chronic variability of anaphylaxis, the persistent burden of mediator symptoms, and the mental health impact of living with a rare and often misunderstood disease contribute to considerable mental health morbidity. Formal psychiatric evaluation and antidepressant therapy should be considered early in the clinical course, with integration of psychiatric support into the MDT framework. Similarly, hepatosplenomegaly and lymphadenopathy, when present, require gastroenterological and hematological assessment, with imaging and, where appropriate, liver biopsy, given the possibility to signal more advanced or aggressive disease.

The systematic assessment of symptom burden and health-related quality of life (HRQoL) using validated patient-reported outcome measures is a clinical imperative, not an optional add-on. The Mastocytosis Quality of Life Questionnaire (MQLQ) and the Mastocytosis Symptom Assessment Form (MSAF) provide structured, reproducible frameworks for covering the full spectrum of SM-related morbidity across domains, including skin symptoms, mediator release episodes, fatigue, and psychological health [49,50]. These instruments should be administered at baseline and at regular follow-up intervals, as longitudinal trends in scores inform treatment escalation decisions and serve as objective metrics for evaluating therapeutic response, including response to novel targeted therapies such as midostaurin and avapritinib in advanced SM.

Finally, shared decision-making must be recognized as an integral clinical competency in SM management. The complexity of treatment choices demands that clinicians actively involve patients, transparently communicate the benefits and harms of available options, and anchor decisions in individual patient values and preferences. Living well with SM requires not only pharmacological expertise but also a sustained partnership between the patient and a coordinated, compassionate, and knowledgeable multidisciplinary team.

## 15. Conclusions

Systemic mastocytosis has evolved from a poorly understood clinical curiosity into a molecularly defined myeloid neoplasm with a clear central driver, *KIT* D816V, and an increasingly precise diagnostic and therapeutic framework. The 2022 WHO and ICC classifications now allow clinicians to integrate histological, immunophenotypic, biochemical, and molecular criteria into a reproducible diagnosis and to stratify patients into non-advanced and advanced subtypes that carry markedly different prognoses and treatment intensities. This precision has been matched on the therapeutic side: the field has progressed from non-selective cytoreductive agents and the multikinase inhibitor midostaurin toward rationally designed, selective type I *KIT* D816V inhibitors—avapritinib and the next-generation agents bezuclastinib and elenestinib—that achieve deeper and more durable disease control with more favorable safety profiles. At the same time, symptom-directed antimediator therapy remains the foundation of care for most patients with indolent disease, and recognition of co-occurring myeloid mutations (*TET2*, *SRSF2*, *ASXL1*, *RUNX1*) is reshaping how advanced SM and SM-AHN are risk-stratified and treated, including growing interest in rational combination regimens and, in eligible patients, allogeneic stem cell transplantation. Important gaps remain: diagnosis is still missed or delayed in patients with atypical presentations (normal tryptase, atypical immunophenotype, or coexisting hereditary alpha-tryptasemia), head-to-head comparisons between the newer *KIT* inhibitors are lacking, and the long-term impact of clonal co-mutations on resistance to *KIT*-selective therapy is not yet fully defined. Addressing these gaps will require continued collaboration between hematologists, allergists, dermatologists, and pathologists, ideally within the expert-center networks (ECNM, AIM) that already coordinate much of the clinical and translational research in this field. For the practicing clinician, the essential takeaway is that systemic mastocytosis is now a treatable, molecularly targetable disease, and that early recognition, accurate subclassification, and timely referral are what translate this scientific progress into better outcomes for patients.

## Figures and Tables

**Table 1:** Pediatric- vs. Adult-Onset Mastocytosis.

Feature/Characteristic	Pediatric	Adult
Most frequent category	Cutaneous mastocytosis	Indolent systemic mastocytosis
Course of disease	Usually resolves	Chronic
Anaphylaxis frequency (%)	<10	50
Tryptase level (µg/L)	<20	Often >20
*KIT* mutation	Exons 8,9,11,17 (40%), absent	D816V (exon 17) (>85–90%)
Morphology of cutaneous lesions	Polymorphic	Monomorphic
Size of lesions	Variable	Small
Distribution	Trunk, head, extremities	Thigh, trunk
Approximately 15% of patients with SM have tryptase < 20 µg/L, and around 10% have normal tryptase levels	Approximately 15% of patients with SM have tryptase < 20 µg/L, and around 10% have normal tryptase levels	Approximately 15% of patients with SM have tryptase < 20 µg/L, and around 10% have normal tryptase levels

**Table 2:** Differential Diagnosis.

Other Mast Cell Disorders	Other Conditions to Consider
Monoclonal mast cell activation syndrome	Dermatologic: chronic spontaneous urticaria, atopic dermatitis, rosacea
Idiopathic mast cell activation syndrome	Neurologic: seizures, stroke, multiple sclerosis, dysautonomia
Anaphylaxis	Psychological: anxiety/panic attacks
	Gastrointestinal: inflammatory bowel disease, irritable bowel syndrome, Zollinger–Ellison syndrome
	Endocrine: thyroid disease, adrenal insufficiency, carcinoid syndrome, VIPoma

**Table 3:** Diagnostic Criteria for Systemic Mastocytosis.

Criterion	Definition
Major	Multifocal dense aggregates of mast cells (≥15 cells) in bone marrow and/or extracutaneous organs
Minor × 4	(1) ≥25% of mast cells with atypical morphology; (2) aberrant CD2, CD25, and/or CD30 expression; (3) *KIT* D816V or other activating *KIT* mutation, particularly when detected in an extracutaneous tissue such as bone marrow (a key feature distinguishing SM from CM); (4) baseline serum tryptase > 20 ng/mL (in absence of associated myeloid neoplasm)

**Table 4:** Identifying Systemic Mastocytosis Subtypes.

SM Variant	Category	B/C-Findings	Key Features
Indolent SM (ISM)	Nonadvanced	≤1 B-finding	Typical skin lesions; most common adult subtype; near-normal survival
Bone Marrow Mastocytosis (BMM)	Nonadvanced	None	No skin lesions; tryptase < 125 ng/mL; no extramedullary dense infiltrates
Smoldering SM (SSM)	Nonadvanced	≥2 B-findings	No C-findings; higher burden than ISM; monitoring essential
SM-AHN	Advanced	Variable	Meets WHO criteria for associated hematological neoplasm (e.g., MDS, MPN, AML)
Aggressive SM (ASM)	Advanced	≥1 C-finding	Organ damage from MC infiltration; requires cytoreductive therapy
Mast Cell Leukemia (MCL)	Advanced	Variable	≥20% MC in BM smears; ≥10% circulating MC (classic) or <10% (aleukemic); poor prognosis

B-findings: higher SM burden (high MC burden, non-MC cytopenia, hepatomegaly, splenomegaly, lymphadenopathy). C-findings: organ damage due to MC infiltration.

**Table 5:** *KIT*-Targeted Tyrosine Kinase Inhibitors in Systemic Mastocytosis.

**Imatinib**	Adult patients with aggressive SM without the *KIT* D816V or with unknown *KIT* status Drug of choice for well-differentiated SM	In a review of 28 patients with ASM: 29% achieved complete response 32% achieved partial response [19]	No contraindications Diarrhea, nausea, ascites, muscle cramps, dyspnea, fatigue Fetal harm can occur
**Midostaurin**	Advanced SM (aggressive SM, SM-AHN, MCL)	Reduced MC infiltration and serum tryptase Improved symptoms and QoL [20]	No contraindications Warnings: Embryofetal toxicity Pulmonary toxicity Gastrointestinal reactions in >50% of patients
**Avapritinib**	Advanced SM (ASM, SM-AHN, MCL)—200 mg QD Indolent SM (ISM) with moderate-to-severe symptoms—25 mg QD	AdvSM (PATHFINDER): ORR 75%, including 19% CR; median OS 62 months at 4-year follow-up ISM (PIONEER): significant improvement in total symptom score vs. placebo ≥50% reduction in serum tryptase in 93% of AdvSM patients [21,22,23,24]	Periorbital edema (69%), anemia (55%), nausea (44%), thrombocytopenia (44%) Intracranial bleeding risk (higher with severe thrombocytopenia) Embryofetal toxicity Cognitive/CNS effects (brain-penetrant)
**Elenestinib**	ISM with inadequate symptom control on BSC (HARBOR phase 2/3 trial) AdvSM and SM-AHN (AZURE phase 1/2 trial)	Next-generation *KIT* D816V inhibitor with limited CNS penetration HARBOR part 1: dose-dependent reductions in serum tryptase (−50.9% at 50 mg; −68.4% at 100 mg vs. +3.3% placebo) Improvements in disease burden biomarkers and symptom scores across all doses [25,26,27,28,29,30]	Well-tolerated at all dose levels (median 35.3 weeks) No treatment-related serious AEs No AEs leading to discontinuation Most AEs grade 1–2; no grade 4/5 events reported
**Bezuclastinib**	NonAdvSM (ISM, SSM, BMM)—SUMMIT trial (100 mg QD) AdvSM (ASM, SM-AHN, MCL)—APEX trial (150 mg QD)	SUMMIT (NonAdvSM): significant symptom improvement vs. placebo (LS mean TSS −24.3 vs. −15.4; *p* = 0.0002) 87.4% achieved ≥50% reduction in serum tryptase; 75.6% in BM mast cells APEX part 1 (AdvSM): ORR 52% by mIWG criteria; 83% ORR at 100 mg BID [31,32,33,34,35]	Most TEAEs are low-grade (70% grade 1) and reversible Most common: hair color change (69.5%), altered taste (23.7%), nausea (22%) Transient ALT/AST elevations; no serious hepatic AEs No bleeding or cognitive impairment events Low CNS penetration profile

SM = systemic mastocytosis; ASM = aggressive SM; ISM = indolent SM; SSM = smoldering SM; BMM = bone marrow mastocytosis; SM-AHN = SM with associated hematologic neoplasm; MCL = mast cell leukemia; NonAdvSM = non-advanced SM; AdvSM = advanced SM; MC = mast cell; ORR = overall response rate; CR = complete remission; OS = overall survival; BM = bone marrow; TSS = total symptom score; QoL = quality of life; BSC = best supportive care; AE = adverse event; TEAE = treatment-emergent AE; CNS = central nervous system; QD = once daily; BID = twice daily.

**Table 6:** Treatment for Mast Cell Mediator-Related Symptoms.

Involved Organ/Symptoms	Stepwise Treatment
Skin: pruritus, urticaria, flushing, angioedema, dermatographism	H1 and H2 blockers Leukotriene receptor antagonist Aspirin Ketotifen Topical cromolyn sodium
GI: abdominal cramping, nausea, vomiting, diarrhea	H2 blockers Cromolyn sodium Leukotriene receptor antagonist Ketotifen Proton pump inhibitors
Neurologic: poor concentration and memory, headache, brain fog	H1 and H2 blockers Aspirin Cromolyn sodium Ketotifen
CV: presyncope, tachycardia	H1 and H2 blockers Corticosteroids Omalizumab
Pulmonary: wheezing, swollen throat	H1 and H2 blockers Corticosteroids Omalizumab Bronchodilators (LABA)
Naso-ocular: nasal stuffiness and pruritus, conjunctival injection	H1 blockers Corticosteroids Cromolyn sodium

## Data Availability

No new data were created or analyzed in this study. Data sharing is not applicable to this article.

## Abbreviations

2-CdA	cladribine
AE	adverse event
AIM	American Initiative in Mast Cell Diseases
AML	acute myeloid leukemia
ASM	aggressive systemic mastocytosis
AHN	associated hematologic neoplasm
AdvSM	advanced systemic mastocytosis
BID	twice daily
BM	bone marrow
BMM	bone marrow mastocytosis
BSC	best supportive care
*CBL*	Casitas B-lineage lymphoma proto-oncogene
CHIP	clonal hematopoiesis of indeterminate potential
CM	cutaneous mastocytosis
CNS	central nervous system
CR	complete remission
DXA	dual-energy X-ray absorptiometry
ECNM	European Competence Network on Mastocytosis
*FLT3*	FMS-like tyrosine kinase 3
HRQoL	health-related quality of life
HαT	hereditary alpha-tryptasemia
IBD	inflammatory bowel disease
IBS	irritable bowel syndrome
ICC	International Consensus Classification
IFN-α	interferon-alpha
ISM	indolent systemic mastocytosis
ISM-SAF	Indolent Systemic Mastocytosis Symptom Assessment Form
LABA	long-acting beta-agonist
MC	mast cell
MCAS	mast cell activation syndrome
MCL	mast cell leukemia
MDT	multidisciplinary team
MDS	myelodysplastic syndrome
mIWG	modified International Working Group
MPN	myeloproliferative neoplasm
MQLQ	Mastocytosis Quality of Life Questionnaire
MSC	mast cell sarcoma
MSAF	Mastocytosis Symptom Assessment Form
NGS	next-generation sequencing
NonAdvSM	non-advanced systemic mastocytosis
ORR	overall response rate
OS	overall survival
PCR	polymerase chain reaction
PDGFR	platelet-derived growth factor receptor
QD	once daily
QoL	quality of life
RANK	receptor activator of nuclear factor kappa-B
SCF	stem cell factor
SDM	shared decision-making
SDT	symptom-directed therapy
SM	systemic mastocytosis
SM-AHN	systemic mastocytosis with associated hematologic neoplasm
SSM	smoldering systemic mastocytosis
TEAE	treatment-emergent adverse event
*TPSAB1*	tryptase alpha/beta 1 gene
TSS	total symptom score
VAF	variant allele frequency
VEGFR	vascular endothelial growth factor receptor
WHO	World Health Organization
